# NIAM-Deficient Mice Are Predisposed to the Development of Proliferative Lesions including B-Cell Lymphomas

**DOI:** 10.1371/journal.pone.0112126

**Published:** 2014-11-13

**Authors:** Sara M. Reed, Jussara Hagen, Viviane P. Muniz, Timothy R. Rosean, Nick Borcherding, Sebastian Sciegienka, J. Adam Goeken, Paul W. Naumann, Weizhou Zhang, Van S. Tompkins, Siegfried Janz, David K. Meyerholz, Dawn E. Quelle

**Affiliations:** 1 Department of Pharmacology, University of Iowa, Iowa City, Iowa, United States of America; 2 Medical Scientist Training Program, University of Iowa, Iowa City, Iowa, United States of America; 3 Molecular and Cellular Biology Program, University of Iowa, Iowa City, Iowa, United States of America; 4 Interdisciplinary Program in Immunology, University of Iowa, Iowa City, Iowa, United States of America; 5 Department of Pathology, University of Iowa, Iowa City, Iowa, United States of America; Virginia Commonwealth University, United States of America

## Abstract

Nuclear Interactor of ARF and Mdm2 (NIAM, gene designation *Tbrg1*) is a largely unstudied inhibitor of cell proliferation that helps maintain chromosomal stability. It is a novel activator of the ARF-Mdm2-Tip60-p53 tumor suppressor pathway as well as other undefined pathways important for genome maintenance. To examine its predicted role as a tumor suppressor, we generated *NIAM* mutant (*NIAM^m/m^*) mice homozygous for a β-galactosidase expressing gene-trap cassette in the endogenous gene. The mutant mice expressed significantly lower levels of NIAM protein in tissues compared to wild-type animals. Fifty percent of aged *NIAM* deficient mice (14 to 21 months) developed proliferative lesions, including a uterine hemangioma, pulmonary papillary adenoma, and a Harderian gland adenoma. No age-matched wild-type or *NIAM^+/m^* heterozygous animals developed lesions. In the spleen, *NIAM^m/m^* mice had prominent white pulp expansion which correlated with enhanced increased reactive lymphoid hyperplasia and evidence of systemic inflammation. Notably, 17% of *NIAM* mutant mice had splenic white pulp features indicating early B-cell lymphoma. This correlated with selective expansion of marginal zone B cells in the spleens of younger, tumor-free *NIAM*-deficient mice. Unexpectedly, basal p53 expression and activity was largely unaffected by NIAM loss in isolated splenic B cells. In sum, *NIAM* down-regulation *in vivo* results in a significant predisposition to developing benign tumors or early stage cancers. These mice represent an outstanding platform for dissecting NIAM's role in tumorigenesis and various anti-cancer pathways, including p53 signaling.

## Introduction

The p53 tumor suppressor forms the core of an extensive signaling network that protects cells against genomic instability and neoplastic transformation in response to genotoxic insults [1]–[3]. Once activated, p53 transactivates or represses a wide array of genes that cause cell cycle arrest, promote DNA repair, restrict metabolism or kill irreparably damaged cells, among other anti-cancer activities [4]. Loss of p53 function occurs in the vast majority of human cancers, if not all, due to *TP53* gene mutation or alteration of its many regulators and targets [2], [5]. Mouse models that lack p53 or express naturally occurring p53 mutants are highly tumor prone and develop the broad range of malignancies found in humans with impaired p53 signaling [6]–[8]. Understanding how p53 activity is controlled, and the importance of its regulators in tumor biology, has been a top priority in cancer research for more than two decades [1]–.

NIAM (Nuclear Interactor of ARF and Mdm2, also called *Tbrg1*) is a new p53 regulator and anti-proliferative factor whose role in cancer is not yet defined [10]–[13]. Mechanistically, it engages the p53 pathway at multiple levels. First, NIAM promotes the relocalization of ARF (Alternative Reading Frame protein), a major activator of p53 [14], from nucleoli into the nucleoplasm where it is more effective at activating p53 [10], [15], [16]. Second, NIAM binds to Mdm2, the primary antagonist of p53 [17], and reduces the formation of p53-Mdm2 complexes [10], [12]. As such, NIAM impairs Mdm2-mediated polyubiquitylation and proteasomal degradation of p53, thereby stabilizing and activating p53 signaling [12]. Third, NIAM is a chromatin-bound protein that associates with the histone acetyltransferase, Tip60 (Tat-interacting protein of 60 kDa) [12], which is essential for p53-dependent cell cycle arrest and death [18]–[20]. Tip60 knockdown studies demonstrated that it contributes to NIAM-mediated p53 activation, revealing that NIAM induces p53 activity through multiple mechanisms involving Tip60 association as well as Mdm2 inhibition [12]. Notably, like p53 and Tip60, NIAM is normally kept at low expression levels in cells by Mdm2-dependent ubiquitylation [10].

The interplay of NIAM with p53 and its established partners (ARF, Mdm2 and Tip60), all of which are frequently disrupted in human cancers [3], [14], [17], [21], [22], suggests NIAM may have tumor suppressive activity. That idea is supported by *in silico* evidence from microarray databases suggesting significant down-regulation of NIAM mRNA levels in many advanced human cancers [23], [24]. Interestingly, NIAM can act independently of ARF-Mdm2-p53 signaling. It can inhibit proliferation in cells lacking ARF, Mdm2 and/or p53, and its depletion in *ARF/Mdm2/p53*-null mouse embryo fibroblasts results in chromosomal instability [10]. These findings imply that NIAM plays an important role in other anti-cancer pathways outside of the ARF-Mdm2-p53 tumor suppressor pathway.

Here, we describe the generation and initial characterization of the first *NIAM* mutant mouse model. These animals have hypomorphic *NIAM* alleles that result in greatly impaired expression of NIAM protein in tissues, similar to what may occur in human malignancies in which its mRNA expression is down-regulated. Spontaneous tumor formation was assessed and NIAM down-regulation found to increase tumor susceptibility in aged animals. B-cell lymphoma was among the tumors identified and this correlated with a marked expansion of splenic marginal zone B cells in young, tumor-free *NIAM*-deficient mice. Interestingly, p53 inactivation in B cells promotes splenic marginal zone B cell expansion and B-cell lymphoma [25], [26], implicating impaired p53 function in the *NIAM* knockout phenotype. At least under non-stressed conditions, however, splenic B cells from young *NIAM*-deficient mice showed no significant effect on basal p53 activity. We suggest that the *NIAM* mutant mice described in this study represent a unique model of B-cell lymphoma that should help resolve NIAM's biological role in p53 signaling and other cancer pathways.

## Results

### Decreased NIAM mRNA expression in human tumors

**Figure 1:**
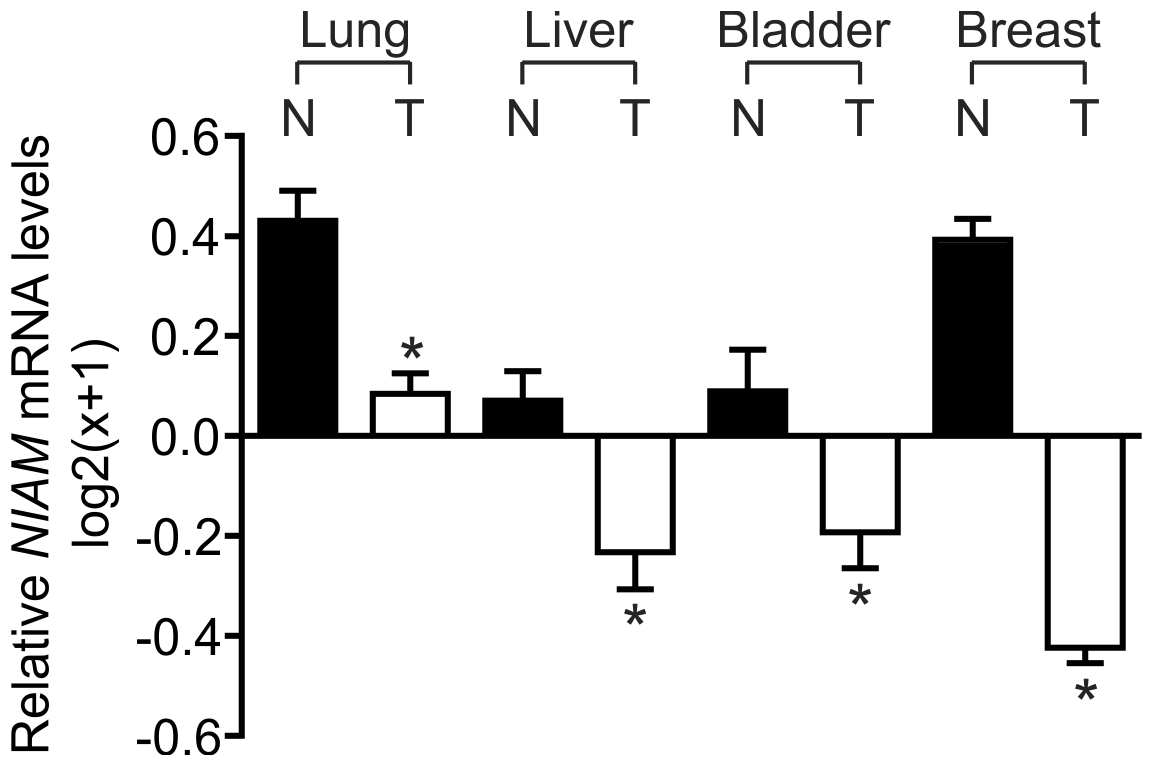
*NIAM* mRNA expression is downregulated in multiple human cancers. *NIAM* mRNA levels are significantly reduced in primary tumor (T) samples relative to normal (N) tissue, according to RNA-seq data obtained from the TCGA project database. The number of samples analyzed for each tissue was as follows: lung (N = 58, T = 488), liver (N = 50, T = 147), bladder (N = 19, T = 211), and breast (N = 108, T = 992). Error bars represent a 95% CI as calculated using the standard error, and an unpaired Welch's T-test was used to calculate statistical significance (*, p<0.0001).

It is well established that ARF-Mdm2-Tip60-p53 signaling plays a dominant role in carcinogenesis [1]–[3], [17], [21], [27]. Since NIAM has important roles in regulating this pathway, we probed various online databases for the most recent information on *NIAM* alterations in human cancers. A previous analysis of the ONCOMINE microarray database in 2007 suggested that *NIAM* mRNA expression is down-regulated in multiple advanced human cancers [10]. The addition of large amounts of microarray results to ONCOMINE since that time only strengthens that conclusion, once again suggesting significant reduction of *NIAM* mRNA levels in many cancers including lung, breast, brain, prostate, and B-cell lymphoma (**Table 1**). Recent RNA sequencing data from The Cancer Genome Atlas (TCGA) project is now available for certain cancers and verifies that there is a marked decrease in *NIAM* (gene name *Tbrg1*) mRNA levels in human lung, liver, bladder, and breast cancers as compared to paired, normal tissues (**Fig. 1**). These data demonstrate that *NIAM* expression is reduced in many different types of human malignancies, consistent with the prediction that it plays an important role in inhibiting tumorigenesis.

**Table 1:** Reduced *NIAM* mRNA levels in cancer tissues.

Tumor Type	No. of Studies*	References
Brain	9	[63]–[67]
B-cell Lymphoma	7	[33]–[37]
Lung	2	[68]
Breast	10	[69]–[71]
Prostate	2	[72]

*Microarray analyses identified through ONCOMINE showing a statistically significant decrease in *NIAM* mRNA in tumors. (p<0.05).

### Generation of NIAM-deficient mice

**Figure 2:**
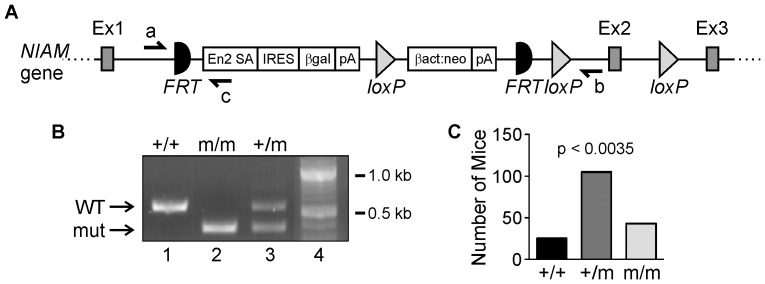
Generating and verifying *NIAM* gene disruption in mice. **A**. The targeted *NIAM* gene locus for the conditional-ready mouse knockout model. Cre-recombinase LoxP sites and Flippase targeted FRT sites are shown, as are locations for the PCR primers to detect the endogenous *NIAM* allele (a+b) or the mutant allele (a+c). Ex, exon. **B**. PCR amplification of the mutant (mut) allele results in a 380 bp product whereas the 548 bp product reflects the wild-type (WT) *NIAM* allele. DNA standards are shown in lane 4. **C**. Number of mice with each genotype (+/+, +/m, m/m) from heterozygous mouse crossings. Chi-square analysis shows a statistically significant difference from the Mendelian distribution of 1∶2∶1.

A *NIAM* gene targeting construct was inserted between exon 1 (which contains the ATG start site) and exon 2 in C57BL/6N embryonic stem cells by the Knockout Mouse Project (KOMP) (**Fig. 2A**). The cassette contains a poly (A) adenylation site, a neomycin resistance gene (Neo), and β-galactosidase trap for tracking normal *NIAM* expression patterns in tissues. It also contains FRT sites for removal of neomycin and β-galactosidase cassettes by flippase, which would restore normal gene function of *NIAM*. Two intronic LoxP sites enable conditional deletion of exon 2 using Cre-expressing mice should the cassette not lead to sufficient loss of the *NIAM* gene. This construct is predicted to interfere with splicing or, at minimum, generate a severely truncated chimeric protein containing only 48 N-terminal residues of NIAM.

We obtained chimeric mice expressing the mutant (m) *NIAM* allele from KOMP and generated NIAM heterozygous (+/m) mice by crossing them to C57BL/6N breeders. After obtaining germline transmission, we interbred heterozygous mice to obtain homozygous offspring (m/m) (**Fig. 2B**). Heterozygous mouse matings (n = 31) produced litter sizes between 1 to 9 pups (avg  = 5.58). The genotype (+/+, +/m, m/m) was determined for 173 mice and the expected Mendelian ratios of 1∶2∶1 were not met (p = 0.0035, using the χ^2^-test) (**Fig. 2C**). The number of heterozygous *NIAM^+/m^* and homozygous *NIAM^m/m^* mice was higher than expected, possibly suggesting that *NIAM* loss may provide a selective advantage during embryogenesis. The *NIAM^m/m^* mice also successfully mated and produced offspring demonstrating no crucial effects of NIAM on fertility and viability.

**Figure 3:**
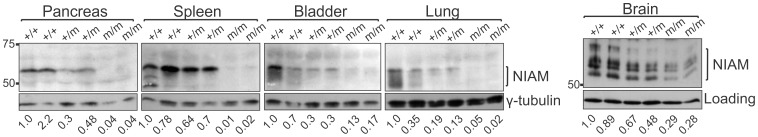
NIAM protein expression in tissues of *NIAM* mutant mice. NIAM protein levels were assessed in mouse tissues from two mice of each genotype (+/+, +/m, m/m). Equivalent amounts of protein lysates from spleen, bladder, lung, brain, and pancreas were analyzed by immunoblotting with antibodies to NIAM and γ-tubulin. The relative expression of NIAM in each sample, as compared to the first wild-type (+/+) sample in each tissue set, is denoted below the lanes. Relative NIAM levels were determined by Image J analysis of band intensities followed by normalization of those values to quantified intensities of the loading control (γ-tubulin or the non-specific band in brain lysates) in each sample.

To determine if the gene-trap cassette effectively interfered with *NIAM* expression, mouse tissues from each genotype were obtained and assessed for levels of NIAM protein. NIAM expression was greatly diminished although some level of expression remained in all *NIAM^m/m^* mouse tissues examined, demonstrating that these animals have hypomorphic *NIAM* alleles (**Fig. 3**). Effective down-regulation of NIAM expression was observed in the bladder, lung, pancreas and spleen of homozygous m/m mice. By comparison, protein extracts from the brain (**Fig 3**) and testes (not shown) of the *NIAM^m/m^* mice showed decreased yet substantial remaining expression of NIAM as compared to wild-type mice. Interestingly, many bands for NIAM protein were detected in some tissues, such as brain, consistent with database predictions for the existence of numerous alternative transcripts of *NIAM*. Tissues were also examined for expression of p53, Mdm2 and p21. NIAM would be predicted to reduce their levels; however, we did not expect to detect significant changes in their expression since p53 signaling is generally kept off in normal tissues under non-stressed conditions. Indeed, basal expression of all three factors was low, and in some cases undetectable, in whole tissue lysates with no consistent differences observed between *NIAM^m/m^* and wild-type mice (data not shown). Immunohistochemical analyses of tissues to identify cell-specific changes in naïve versus stressed mice exposed to p53 activating stimuli (e.g., irradiation) should more effectively reveal effects of NIAM down-regulation on the p53 pathway *in vivo*. Overall, a significant decrease in NIAM expression was observed in *NIAM^+/m^* and *NIAM^m/m^* mice relative to wild-type controls, indicating these animals are a useful model for examining the biological effects of reduced NIAM expression *in vivo*.

### Loss of NIAM *in vivo* promotes spontaneous tumor formation

**Figure 4:**
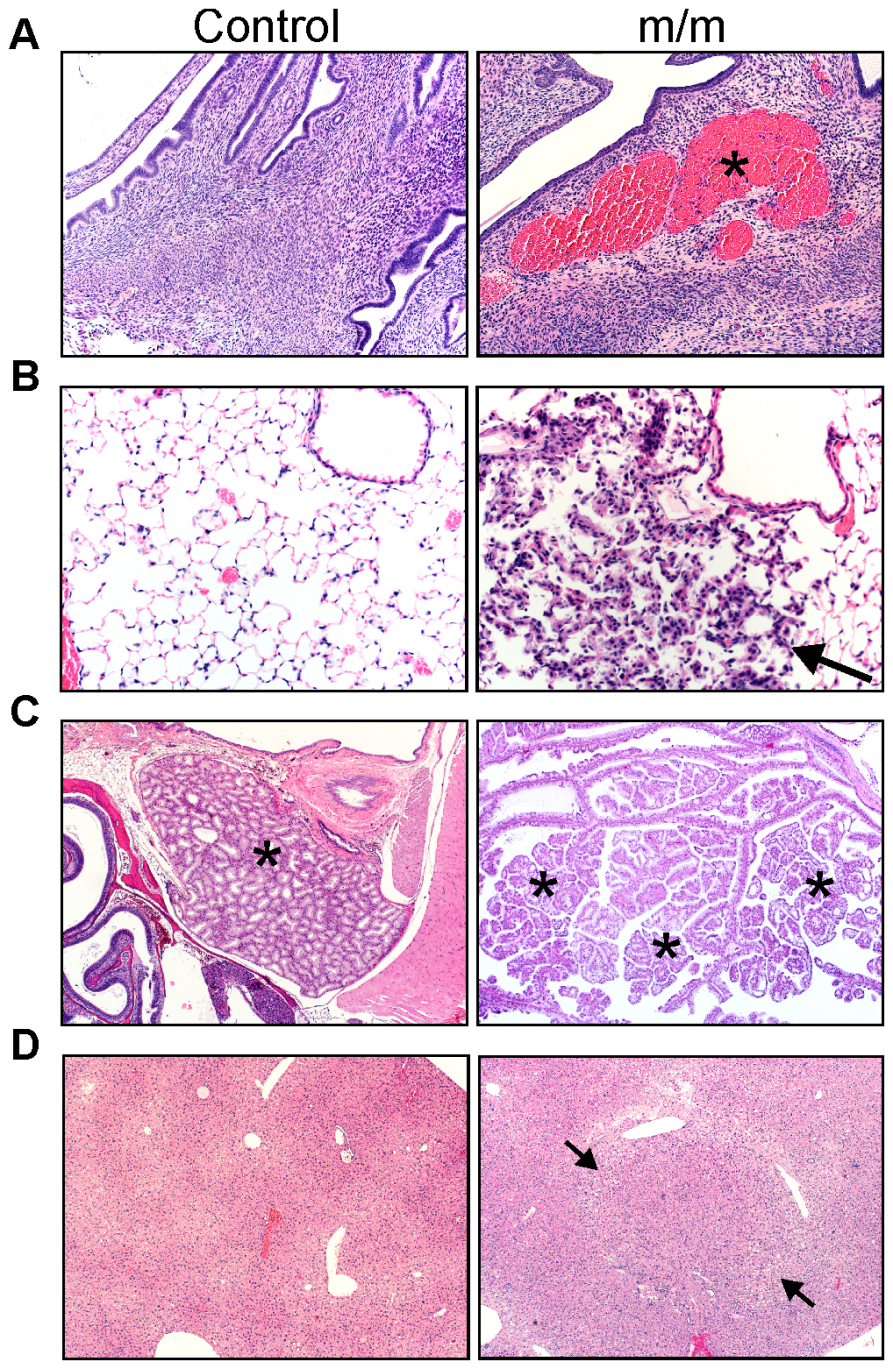
*NIAM^m/m^* mice are predisposed to proliferative lesions. Histopathologic analyses of multiple mouse tissues. **A**. A hemangioma (asterisk, right panel) found in the uterus of a *NIAM^m/m^* mouse compared to normal uterine tissue (left panel). (H&E, 100x) **B**. Pulmonary papillary adenoma in a homozygous *NIAM^m/m^* mouse (arrow, right panel). A depiction of normal lung tissue from a control mouse is shown (left panel). (H&E, 20x) **C**. Harderian gland adenoma of a *NIAM^m/m^* mouse (asterisks, right panel). A representative image of a normal Harderian gland (asterisk) from a control mouse is depicted (left panel). (H&E, 40x) **D**. A representative image of a normal liver from a control mouse (left panel) versus a focus of cellular alteration (arrows) in a *NIAM^m/m^* mouse (right panel). (H&E, 40x) Controls represent tissues from similarly aged wild-type (panels B and C) and *NIAM^+/m^* heterozygous (panels A and D) mice, which were found to be indistinguishable in our analyses.

*NIAM^m/m^* mice grow to adulthood and normal size, are fertile, and are externally indistinguishable from wild-type mice. Moreover, full pathological examination of young *NIAM* mutant animals at approximately 8 weeks of age showed no apparent tissue abnormalities, suggesting that tissue development is unaffected by *NIAM* deficiency. However, NIAM carries out a variety of anti-cancer activities in cultured cells [10], [12] and our database analyses (**Table 1** and **Fig. 1**) indicate its expression is down-regulated in many human cancers, suggesting it may normally prevent tumor development. Thus, we assessed aged mice of all three *NIAM* genotypes for spontaneous tumorigenesis. As shown in **Table 2**, 50% of *NIAM^m/m^* mice developed a neoplastic lesion whereas no wild-type and heterozygous mice of similar age showed obvious signs of tumorigenesis (*p* = 0.0025, Fisher's exact test). The *NIAM^m/m^* mice developed different types of benign tumors including a uterine hemangioma (**Fig. 4A**), an early lung papillary adenoma (**Fig. 4B**), and a Harderian gland adenoma (**Fig. 4C**). In addition, a focus of cellular alteration (FCA) was seen in the liver of one *NIAM^m/m^* mouse (**Fig. 4D**), a precancerous lesion seen in other mouse models [28]. These histopathological findings suggest that NIAM normally suppresses neoplasia.

**Table 2:** Neoplastic lesions in *NIAM*-deficient mice.

Genotype	Avg. Age at Necropsy (weeks)	# of mice with Neoplasms*	Types of neoplasms
*NIAM^+/+^*	68.2	0/8	-
*NIAM^+/m^*	68.5	0/8	-
*NIAM^m/m^*	66	6/12	Early B-cell lymphoma (2) Uterine hemangioma (1) Preputial gland cyst (1) Harderian gland adenoma (1) Lung adenoma (1)

*, p<0.0025 by Fisher's Exact Test between control (*NIAM^+/+^* and *NIAM^+/m^)* mice and *NIAM^m/m^* mice.

### Inflammation and early B-cell lymphoma develop in *NIAM^m/m^* mice

**Figure 5:**
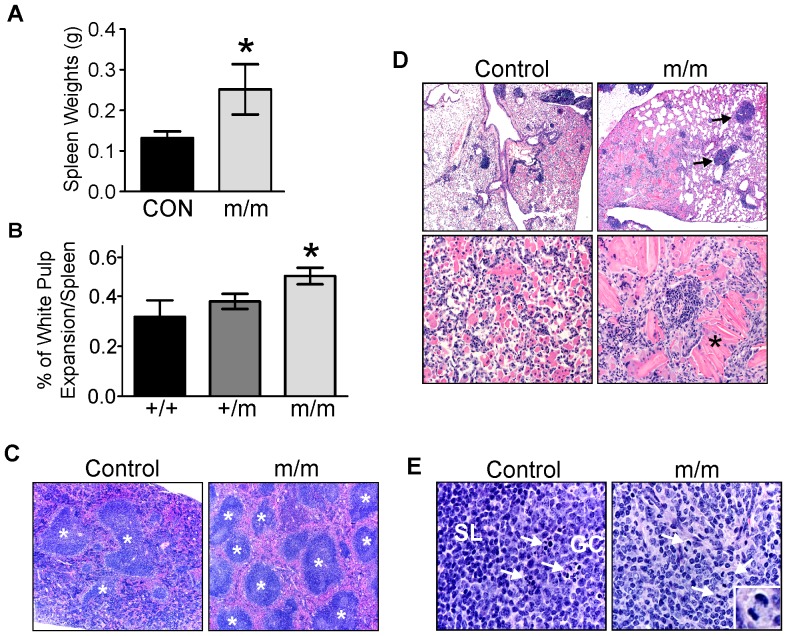
Loss of NIAM promotes reactive lymphoid hyperplasia and B-cell lymphoma in the spleen. **A**. Weights of spleens from +/+ and +/m mice (CON, n = 15) compared to *NIAM^m/m^* mice (m/m, n = 12). Note: one WT (+/+) mouse with an incidental case of severe chronic pyometra was excluded from the analysis. Statistical significance was shown by a Mann-Whitney test (*, p<0.05) **B**. The percentage of splenic white pulp per total splenic area was calculated for each genotype. *NIAM^m/m^* mice (n = 8) are statistically different from both wild-type (+/+, n = 5) and heterozygous (+/m, n = 6) mice as calculated by an unpaired two-tailed T-test. (*, p<0.05) Note: one WT (+/+) and one mutant (m/m) mouse were excluded from the analysis because extensive extramedullary hematopoiesis prevented accurate visualization of the white pulp **C**. Representative images of spleens from control wild-type (+/+) and homozygous m/m mice (H&E, 40x). Splenic white pulp (asterisks) was increased in *NIAM^m/m^* mice. Note: these samples show a range of variation in the extent of red pulp extramedullary hematopoiesis that was seen for both groups. **D**. Eosinophilic crystalline pneumonia in the lungs of control *NIAM^+/m^* heterozygous and *NIAM^m/m^* mice. Loss of NIAM resulted in more severe disease with extensive pulmonary lymphoid inflammation (arrows) and large extracellular crystals (asterisks) in the affected *NIAM^m/m^* mouse. (H&E, 40× top and 100× bottom panels) **E**. White pulp from control *NIAM^+/m^* heterozygous and *NIAM^m/m^* homozygous mice (H&E, 600x). A spleen from a heterozygous mouse with chronic ulcerative dermatitis and reactive hyperplasia of white pulp characterized by a germinal center (GC) and tingible body macrophages full of cellular debris (arrows) on a background of small lymphocytes (SL) (left panel). In two homozygous m/m mice, typical white pulp architecture was multifocally depleted and effaced by centroblasts and centrocytes with multiple mitotic figures (inset and arrows) (right panel). Note that *NIAM^+/m^* heterozygous and *NIAM^+/+^* wild-type mice appeared identical in our analyses and were therefore used interchangeably as controls.

A prominent finding within the cohort was the pathological changes in the spleens of older *NIAM* mutant mice, which were significantly increased in size compared to wild-type and heterozygous mouse spleens (p <0.05) (**Fig. 5A**). Hematoxylin and Eosin (H&E) staining showed that NIAM deficiency led to a significant expansion of the splenic white pulp compared to control age matched cohorts (**Fig. 5B, C**). These white pulp changes in *NIAM^m/m^* mice were often due to reactive hyperplasia. This, along with perivascular lymphoid aggregates in various visceral organ, was consistent with systemic inflammation in mice [29]. In addition, one *NIAM^m/m^* mouse had severe diffuse eosinophilic crystalline pneumonia (ECP) whereas only one control had minor localized ECP disease (**Fig. 5D**). ECP may be seen in certain strains of mice including those with immunodeficiency and Th2 prone inflammatory environments [30], [31]. Altogether, these data suggest that NIAM mutant mice have a proinflammatory phenotype. Additionally, two of the twelve (17%) *NIAM^m/m^* mice developed early B-cell lymphoma. Their spleens had multifocal loss of the white pulp architecture (e.g. small lymphocytes, germinal centers and tingible body macrophages) with replacement by highly mitotic centrocytic and centroblastic cells (**Fig. 5E** **)** [32]. When considered with the development of various benign neoplasms in other *NIAM^m/m^* animals, these results indicate that NIAM expression is required to prevent spontaneous tumorigenesis.

### Marginal zone B cells are increased in *NIAM^m/m^* mice

**Figure 6:**
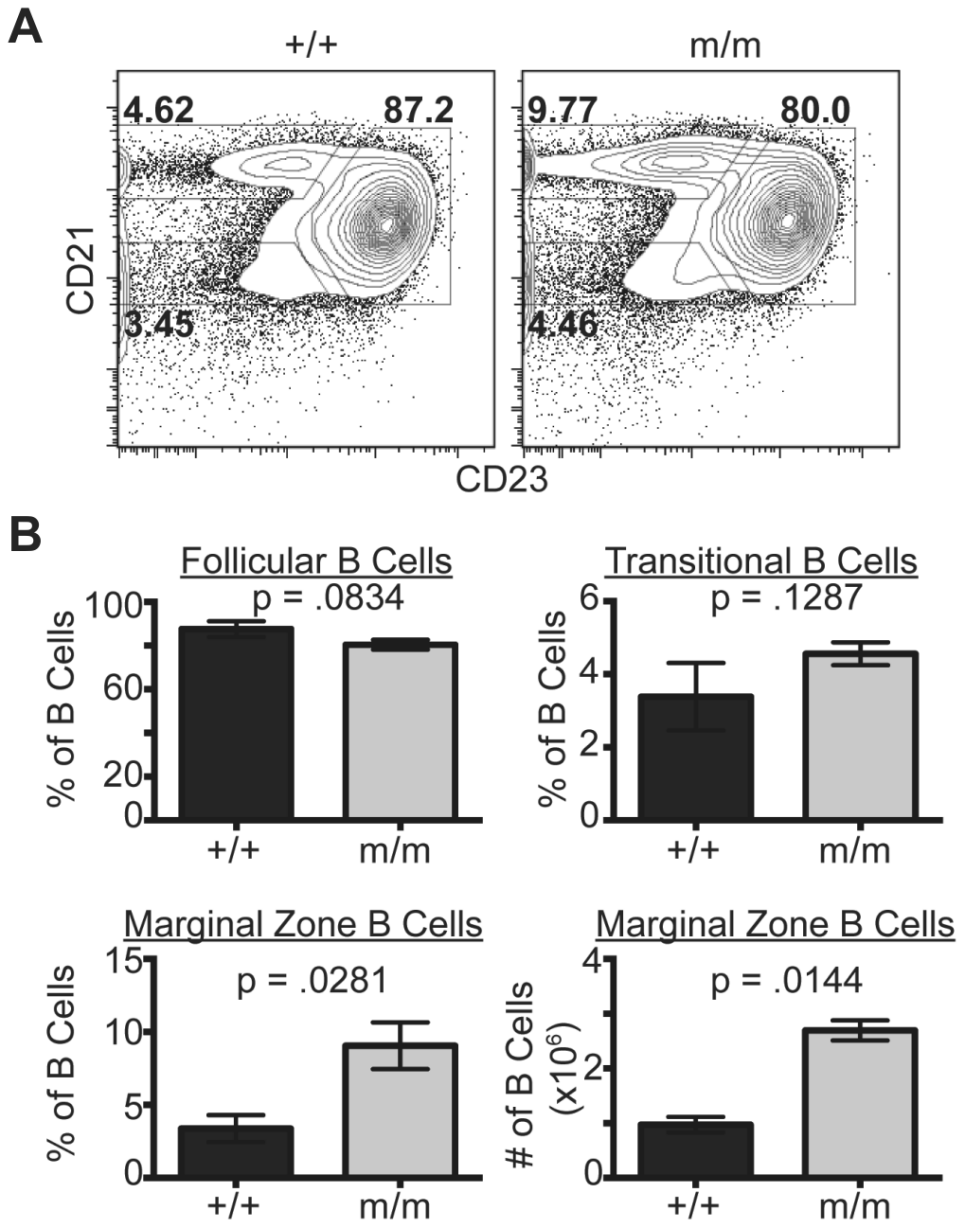
Marginal zone B cells are increased in *NIAM^m/m^* mice. Flow cytometric analyses of splenic B cell development. Splenic B cells were isolated from young, 6 month old tumor-free wild-type (+/+) and *NIAM* mutant (m/m) mice (three of each genotype). **A**. Representative flow cytometry plots of *NIAM* wild-type versus m/m IgM positive splenocytes. Transitional (CD21^lo^/CD23-), follicular (CD21+/CD23+) and marginal zone (CD21^hi^/CD23-) B cells are identified. **B**. Average frequency of follicular (top left), transitional (top right), and marginal zone (bottom left) B cells from mice of the indicated genotypes. Average marginal zone B cell numbers are also shown (bottom right). All p values were calculated using unpaired, two-tailed Student's T tests.

Since aged *NIAM^m/m^* mice have enlarged spleens and some develop B-cell lymphoma, we evaluated B cell development in younger, tumor-free mice (6 months of age, 3 of each *NIAM* genotype) to identify potential pre-malignant changes that could give rise to B-cell tumors. Analysis of B cell development in the bone marrow of *NIAM^m/m^* mice revealed no alterations compared to wild-type control animals (**Fig. S1**). Specifically, there were no significant differences in the frequency of pro/pre (B220+IgM-, p = 0.4783), immature (B220+IgM+, p = 0.7693) or mature (B220^hi^IgM+, p = 0.8048) B cells. Analysis of B cells from the spleen likewise showed no difference in the frequency of transitional (CD21^lo^/CD23-) or follicular (CD21+/CD23+) B cells (**Fig. 6A, B**; p values of 0.5068 and 0.9325, respectively). In contrast, splenic marginal zone B cells (CD21^hi^/CD23-) were significantly increased in frequency (p = 0.0281) and number (p = 0.0144) in *NIAM^m/m^* mice compared to wild-type controls (**Fig. 6**). These results uncover a specific sensitivity of splenic marginal zone B cells to NIAM loss, which may suggest that NIAM normally restricts their proliferation.

**Figure 7:**
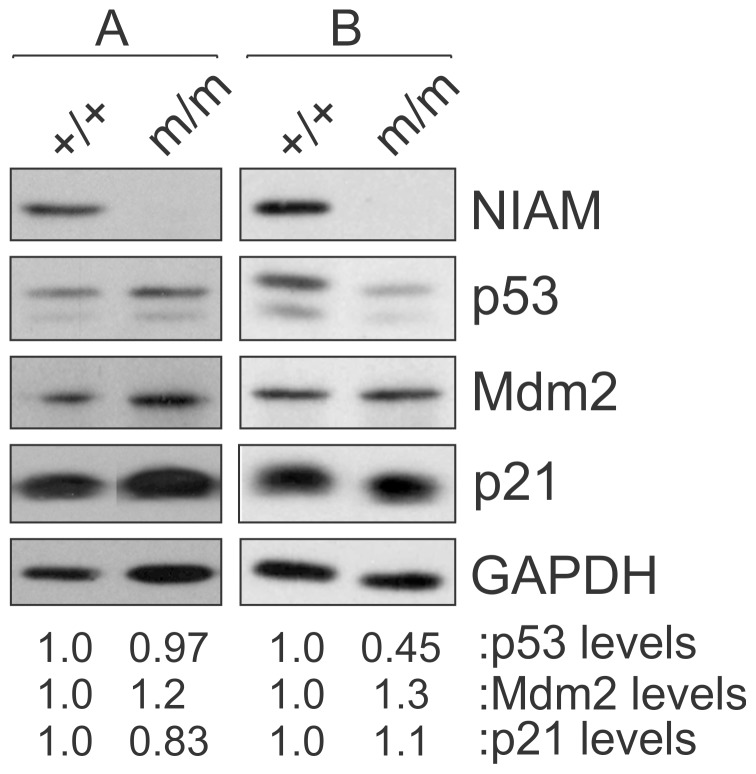
NIAM is not required for basal p53 activity in splenic B cells. Splenic B cells from young, tumor-free wild-type *NIAM^+/+^* or *NIAM^m/m^* homozygous mutant mice were isolated and expression of endogenous NIAM, p53, Mdm2 and p21 proteins was assessed by western blotting. Relative levels of p53, Mdm2 and p21 (normalized to GAPDH loading and compared to the untreated, wild-type control sample) were determined following quantification of bands using Image J. Representative results from 4 of 5 pairs of mice (both 8 week and 6 months of age) are shown in Set A, while Set B shows data for one pair of 6 month old mice. Set A and B samples were analyzed on separate gels, and lanes within each set were spliced together from the same autoradiogram for image clarity.

Selective expansion of splenic marginal zone B cells also occurs in mice with conditional inactivation of p53 in B cells and it is associated with their development of B-cell lymphomas [25], [26]. Since NIAM is a positive regulator of p53 expression and transcriptional activation [12], and some NIAM mice developed B-cell lymphoma, we wondered if the B cell phenotype in *NIAM^m/m^* mice could be associated with reduced p53 signaling. Therefore, we examined p53 status in LPS-stimulated splenic B cells isolated from five wild-type *NIAM^+/+^* and five mutant *NIAM^m/m^* mice. Western analyses showed complete NIAM loss in *NIAM^m/m^* cells compared to wild-type B cells (**Fig. 7**). We expected NIAM loss would reduce p53 levels and activity, but surprisingly it had no effect on basal expression of p53 or its targets, Mdm2 and p21, in the B cells of most *NIAM^m/m^* mice (4 of 5) relative to NIAM-positive B cells (**Fig. 7**, set A). These results were seen in B cells isolated from both 8 week and 6 month old mice, suggesting the marginal zone B cell expansion in 6 month old *NIAM* mutant mice is likely independent of p53. Notably, one *NIAM* mutant mouse had decreased B cell expression of p53 although this did not correlate with reduced expression of Mdm2 or p21, at least under these stress-free conditions (**Fig. 7**, set B). These analyses show that NIAM is not required for basal p53 activity in splenic B cells but may, depending on the context, predispose to p53 down-regulation. Additional studies examining p53 stimulation and checkpoint activation following DNA damage or other genotoxic insults in *NIAM*-deficient B cells are warranted.

## Discussion

This study is the first investigation of NIAM function *in vivo* using newly generated *NIAM*-deficient mice. A tumor suppressor role for NIAM was anticipated given its ability to activate p53 and other currently undefined anti-cancer pathways involved in growth inhibition and genome maintenance [10], [12]. Consistent with that prediction, reduced NIAM expression in mice yielded a tumor phenotype characterized by the development of both benign and cancerous lesions, including early B-cell lymphoma. No neoplasms developed in similarly aged heterozygote or wild-type controls, strongly suggesting that tumor formation was due to NIAM loss. It is noteworthy that systemic inflammation was seen in multiple mice as that often precedes tumorigenesis. Although mice from each genotype (+/+, +/m, m/m) in our cohort showed signs of heightened inflammation, only the *NIAM^m/m^* animals develop lesions. Thus, it is possible that reduced NIAM expression may accelerate or exacerbate the tumor promoting effects of a chronic inflammatory response.

The development of B-cell lymphoma in *NIAM^m/m^* mice is consistent with several observations. First, microarray analyses suggest that *NIAM* mRNA levels are significantly reduced in human B-cell lymphomas [33]–[37]. Second, NIAM's functional partners, ARF, Mdm2, and p53 each play a critical role in that disease. ARF and p53 are commonly inactivated whereas Mdm2 is overexpressed in a majority of B-cell lymphomas in patients [38]–[40], and genetically engineered mice that mimic those alterations effectively model the disease [41]–[43]. Finally, NIAM is a growth inhibitor whose activation of p53 in cultured cells results in induction of *p21* (*CDKN1A/Waf1/Cip1*) [10], [12], a key transcriptional target of p53 [44], [45]. Others previously showed that a significant fraction (14%) of tumors that develop in *p21* knockout mice are B-cell lymphomas [46], which is similar in incidence (17%) to the B-cell lymphomas arising in *NIAM^m/m^* mice.

Our data show that NIAM contributes to tumor suppression but to a lesser extent than p53 or ARF. Half of *NIAM^m/m^* mice developed benign and early cancerous lesions at an average age of 16.5 months (66 weeks). By comparison, mice lacking p53 in all tissues develop a range of malignancies (mainly T-cell lymphomas and sarcomas) with full penetrance at approximately 5 months of age [6], [7]. Less pronounced *in vivo* effects are observed for disruption of p53 regulators and targets relative to loss of p53 itself [2]. For example, animals lacking ARF, a major positive regulator of p53, develop tumors at a slower rate (∼10 months average age) than *p53*-null mice [47], [48]. Among p53 targets, loss of *PUMA* causes no increased susceptibility to tumorigenesis [49] while deletion of *p21* yields a moderate tumor phenotype [46]. Specifically, *p21*-null animals develop tumors at an average age of 16 months and with reduced incidence (27% in females, 55% in males) compared to mice lacking *p53*. Thus, a milder tumor phenotype for *NIAM^m/m^* mice is not surprising since it represents just one of many p53 regulators [1], [3].

We currently know very little about the normal physiological or pathological signals that control NIAM expression and where its function is required. The expression of β-galactosidase in these mutant mice will provide an excellent surrogate for tracking normal patterns of NIAM expression during development, in particular tissue and cell types, and in response to different stimuli. The interplay between NIAM, p53, and ARF, as well as NIAM's regulation of p21 expression, may be instructive. For instance, loss of each factor (p53, ARF, or p21) is associated with impaired stem cell maintenance and renewal for a variety of cellular lineages, including hematopoietic and neuronal cells, suggesting a possible contribution by NIAM to those processes and cell types [50]–[52]. Similarly, one of the most significant cellular stresses that stimulate p53 activity is DNA damage [1]. We recently found that treatment of MCF10A mammary epithelial cells causes NIAM protein upregulation (**Fig. S2**), implying a role for NIAM in the DNA damage response. The importance of NIAM to p53 activation and maintenance of DNA integrity in different tissues in response to DNA damage or other diverse stimuli may now be evaluated in an *in vivo* context.

A link between *NIAM* and *p53* in B cell development and lymphoma was strongly suggested by findings that mice lacking those genes each display splenic marginal zone B cell expansion and B-cell lymphoma ([25], [26] and this study). Yet NIAM loss had minimal effects on basal p53 expression and activity in B cells from young animals, with just one of five *NIAM^m/m^* mice displaying lower p53 levels. That unexpected result suggests the splenic marginal zone B cell enrichment in young *NIAM^m/m^* mice is independent of p53. However, it remains to be determined if p53 function in response to DNA damage or other cellular stresses is diminished in *NIAM^m/m^* cells and contributes to tumor development in older animals. Indeed, it is conceivable that lower basal p53 expression in splenic B cells observed in some *NIAM^m/m^* mice impairs stress-induced p53 signaling and checkpoint activation, ultimately fostering B-cell lymphomagenesis in aging mice. Future studies will assess that possibility.

Crosses between *NIAM* mutant mice with other genetic models of cancer will be instrumental in determining NIAM's contribution to p53 signaling as well as other cancer pathways. In that regard, *in vivo* studies should help resolve the biological relevance of NIAM's association with the ARF tumor suppressor [10]–[12]. Initial studies showed NIAM is capable of mobilizing ARF into the nucleoplasm [10], where ARF is known to more effectively bind Mdm2 and activate p53 [15], [16], yet NIAM and ARF can nonetheless act independent of each other to transiently activate p53 [10], [12]. It is possible that NIAM's inhibition of Mdm2 and association with Tip60 may help sustain ARF-p53 signaling and promote senescence in cells following DNA damage or oncogene activation. If so, this would represent an important anti-cancer function since p53-mediated cellular senescence (induced by ARF or other mechanisms) is thought to play a pivotal role in tumor suppression [53]–[56].

Earlier work on NIAM and much of this Discussion has revolved around the idea that NIAM would prevent tumorigenesis, at least in part, through its effects on p53. However, it is likely that NIAM has significant anti-cancer functions *in vivo* that are independent of p53. One reason is that NIAM can inhibit proliferation and promote chromosomal stability independent of ARF, Mdm2, and p53, indicating it acts in other important pathways relevant to maintaining genomic stability [10]. Moreover, in this study basal p53 expression in splenic B cells was unchanged by NIAM loss in most animals. One factor besides p53 that could contribute to the tumor phenotype in *NIAM^m/m^* mice is transforming growth factor beta 1 (TGF-β1). *NIAM* was originally identified as a TGF-β1 responsive gene [13] and we previously found it is induced in TGF-β1-arrested cells [10]. TGF-β1 is a potent inhibitor of proliferation in many cell types (epithelial, endothelial, hematopoietic, etc), but alterations of its signaling components in neoplastic cells ultimately causes it to drive proliferation and cancer progression [57], [58]. If NIAM is an effector of TGF-β signaling, its down-regulation in *NIAM^m/m^* mice may diminish the anti-proliferative activities of the pathway and consequently enhance its tumor-promoting effects.

Another factor of interest is the nuclear transcription factor, NF-kappa B. NF-κB was originally identified in B cells, contributes to marginal zone B cell formation, and is often constitutively activated in numerous inflammatory conditions and human cancers, including B cell lymphomas [59], [60]. NF-κB is activated by a multitude of stimuli and cross-talks with many essential molecules (e.g., p53 and STAT3) and complex signaling pathways (including TGF-β) that control inflammation, cell survival and cell proliferation, among other key biological processes. The enrichment of marginal zone B cells as well as progressive expansion of splenic white pulp and development of B-cell lymphomas in *NIAM* mutant mice supports the prediction that NF-κB signaling may be hyper-activated when NIAM is down-regulated.

Overall, our data show that *NIAM* deficiency is associated with a proinflammatory phenotype and facilitates spontaneous tumorigenesis. Based on the literature, we speculate that the marginal zone B cell expansion in *NIAM* mutant mice is intimately related to their development of systemic inflammation [61] and B cell lymphomagenesis [25], [26]. We also predict that NIAM, as a regulator of ARF-Mdm2-Tip60-p53 signaling and other undefined pathways affecting maintenance of chromosomal stability, normally cooperates with multiple anti-cancer pathways to suppress tumor development. The *NIAM^m/m^* mice described here are an outstanding model to explore those concepts.

## Materials and Methods

### Animal Husbandry and Ethics Statement

Mice were housed in the University of Iowa Animal Care barrier facility. Mice were kept in rooms with a 12 hour light-dark cycle and free access to water and food. All mouse experiments were conducted according to protocols approved by the University of Iowa Institutional Animal Care and Use Committee (protocol#1204079). All efforts were made to minimize suffering. Mice were euthanized using carbon dioxide inhalation.

### Generation of NIAM Mutant Mice

The Knockout Mouse Project (KOMP) generated male chimeras by injecting embryonic stem cells containing a *NIAM* (*TBRG1)* allele with a β-galactosidase cassette and LoxP sites around Exon2 into C57BL/6 blastocysts (www.komp.org, Project ID: CSD41510). Chimeric mice were then bred with C57BL/6N females to obtain germline transmission of the mutant *TBRG1* allele. To confirm mouse genotypes, the REDExtract-N-Amp Tissue PCR Kit (Sigma Aldrich, XNAT-100RXN) was used to isolate DNA from mouse tails and to perform PCR. PCR genotyping primers were: Primer a: 5′- GGTCAAAGCTGTAAGCATAGAGAGTC -3′; Primer b: 5′- CTTGAGGCTCCTTTCTGGTG -3′; Primer c: 5′- CCAACTGACCTTGGGCAAGAACAT -3′. PCR reactions consisted of 2 µL DNA added to 5 µl RedExtract PCR Master Mix (Sigma Aldrich), 0.8 µl of each primer and 0.6 µL PCR grade water to a total reaction volume of 10 µL. PCR was performed as follows: 95°C for 5 min, 35 cycles of 95°C for 1 min, annealing of primers at 54°C for 1 min, and extension at 72°C for 1.5 min on a PCR Sprint thermal cycler (Thermo Scientific Hybaid).

### Database Analyses

The Cancer Genome Atlas Pan-Cancer analysis project's individual RNAseq datasets were downloaded from the UCSC Cancer Genome Browser (https://genome-cancer.ucsc.edu/). The UCSC Cancer Browser Team utilized level 3 TCGA RNAseq datasets (available at https://tcga-data.nci.nih.gov/tcga/), log2(x+1) transformed, and renormalized to the expression of all available TCGA cancer cohorts. PanCan normalized, log2(x+1) expression for available paired normal and primary tumor samples were compared for each TCGA cancer cohort utilizing an unpaired Welch's T-test.

### Protein Analyses in Mouse Tissues

Mouse tissues were isolated, flash frozen in liquid nitrogen, and crushed into a powder by mortar and pestle. Tissues were lysed with RIPA buffer (50 mM Tris, pH 8.0, 150 mM NaCl, 1% Triton X-100, 0.1% SDS, 0.5% Sodium deoxycholate) supplemented with 1 mM NaF, protease inhibitor cocktail (Sigma, P8340), phosphatase inhibitor cocktail (Sigma, P0044), and 30 µM phenylmethylsulfonyl fluoride for 1 hour on ice. After brief sonication (2×5s pulses), protein lysates were centrifuged at 14,000 rpm for 15 min at 4°C. The concentration of protein for each tissue was assessed by BCA assay (Pierce, Rockford, IL). Equivalent amounts of total cellular protein was loaded and separated by SDS-PAGE, transferred to polyvinylidene difluoride (PVDF) membranes (Millipore), and analyzed by immunoblotting. Proteins were detected on membranes by ECL (Amersham Biosciences) with antibodies against NIAM (rabbit polyclonal antibody at 1.5 µg/ml [10] and mouse monoclonal antibody [clone 11E12] at 1∶5 dilution [62]) and γ-tubulin (Sigma, T6557, mouse monoclonal antibody, 1∶10,000).

### Primary B Cell Analyses

Bone marrow and spleens were harvested from wild-type and NIAM mutant mice (three 6 month old littermate males per genotype) and minced between frosted glass slides to liberate cells. ACK lysis (Lonza, Radnor, PA) was used to lyse red blood cells. For flow cytometric analyses of B cells, one million lymphocytes were washed and resuspended in staining buffer that consisted of balanced salt solution, 5% bovine calf serum and 0.1% sodium azide. Non-specific binding of antibody was blocked using 10 µl rat serum (Jackson Immunoresearch, West Grove, PA) and 10 µg 2.4G2 (BioXCell, West Lebanon, NH). Cells were then incubated on wet ice in the dark with antibodies to B220-PE-Cy7 (6B2, eBioscience, San Diego, CA), CD23-PE (B3B4, eBioscience), CD21-APC (7G6, BD Biosciences, San Jose, CA), and IgM-FITC (RMM-1, Biolegend, San Diego, CA). Samples were run on a BD FACSCANTO II instrument (Becton Dickinson, San Jose, CA) and data were analyzed using FlowJo (Tree Star, Ashland, OR).

For analyses of p53, B cells were isolated from spleens of 6 month and 8 week old wild-type and NIAM mutant mice using B220 micro beads (Miltenvi Biotec, San Diego, CA) according to manufacturer's specifications. Cells were plated at 5×10^5^ c/ml in B-cell media (RPMI, 10% fetal calf serum, 2 mM Glutamine, 1 mM Sodium Pyruvate, 0.055 mM β-mercaptoethanol and 100 units/ml of penicillin with 100 µg/ml streptomycin) supplemented with LPS (Sigma) at 20 µg/ml to maintain viability. After overnight incubation at 37°C, 5% CO_2,_ cells were harvested and lysed directly in 1× SDS-PAGE sample buffer. Samples (1×10^6^ cell equivalents) were subjected to SDS-PAGE and protein expression examined by western blotting as described above using antibodies to p53 (Santa Cruz, Sc-6243 [FL-393] rabbit polyclonal, 1∶200), Mdm2 (2A10 mouse monoclonal, 1∶10), p21 (Calbiochem, PC55 [Ab-5], rabbit polyclonal, 2 µg/ml), and GAPDH (Abcam, Ab8245, mouse monoclonal, 1∶20,000).

### Mouse Necropsy and Histopathological Analyses

Individual organs were harvested from mice euthanized by CO_2_ asphyxiation. A total of 28 mice (8 *NIAM^+/+^*, 8 *NIAM^+/m^* and 12 *NIAM^m/m^*) between the ages of 14 to 21 months were subjected to pathological examination. Organs assessed by macroscopic and histopathological analyses included pancreas, liver, spleen, gastrointestinal tract, reproductive tract for males and females, kidney, urinary bladder, lung, heart, head sections (e.g., eyes, nasal cavity, etc), and sagittal section of the brain (cerebellum, cerebrum and brain stem). Organ weights (e.g., spleen and liver) were collected and then tissues were formalin fixed and paraffin embedded. Tissue sections (∼4 µm) were made from paraffin blocks and stained with hematoxylin and eosin (H&E). Mouse tissues were analyzed and reviewed for abnormal findings by a pathologist. Quantification of the white pulp of individual mouse spleens stained with H&E was measured by Image J software. The splenic white pulp was evaluated as a percentage of the total splenic area in tissue sections.

## Supporting Information

Figure S1 **Early B cell development is not altered in NIAM-deficient mice.** Flow cytometric analysis of early B cell development. **A**. Representative flow cytometry plots of bone marrow lymphocytes from *NIAM* wild-type (+/+) or mutant (m/m) mice. Early (B220+/IgM-), immature (B220+/IgM+) and mature (B220high/IgM+) B cells are identified. **B**. Average frequency of immature (left), mature (middle) and early (right) B cells in *NIAM* +/+ versus m/m mice. No significant differences were observed. For these studies, bone marrow was isolated and analyzed from six mice total (the same analyzed in Figure 6), three of each genotype.
(TIF)

Figure S2 **Sustained DNA damage induces NIAM expression.** Western analyses show that endogenous NIAM protein is induced by DNA damage caused by exposure to doxorubicin (Dxn, 66 ng/mL) for the indicated times (hrs) in human MCF10A mammary epithelial cells. GAPDH levels serve as control for equivalent loading.
(TIF)
