# Supplementary figures and images for: NIAM-Deficient Mice Are Predisposed to the Development of Proliferative Lesions including B-Cell Lymphomas
Source: PLoS One. 2014 Nov 13;9(11):e112126. doi: 10.1371/journal.pone.0112126 (PMC4231569; doi:10.1371/journal.pone.0112126)

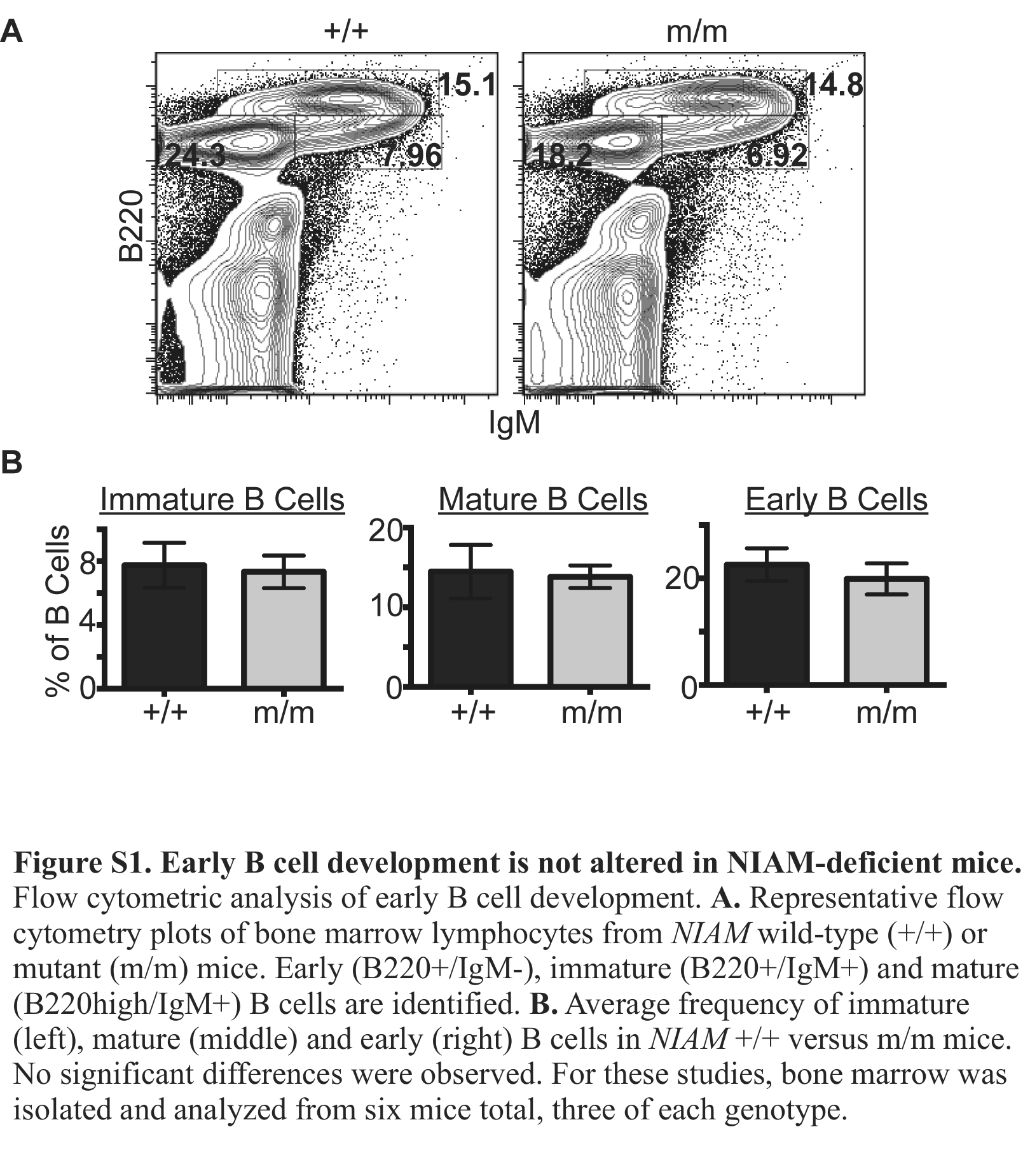

Supplement: Figure S1 — Early B cell development is not altered in NIAM-deficient mice. Flow cytometric analysis of early B cell development. A. Representative flow cytometry plots of bone marrow lymphocytes from NIAM wild-type (+/+) or mutant (m/m) mice. Early (B220+/IgM-), immature (B220+/IgM+) and mature (B220high/IgM+) B cells are identified. B. Average frequency of immature (left), mature (middle) and early (right) B cells in NIAM +/+ versus m/m mice. No significant differences were observed. For these studies, bone marrow was isolated and analyzed from six mice total (the same analyzed in Figure 6), three of each genotype. (TIF) [file pone.0112126.s001.tif]

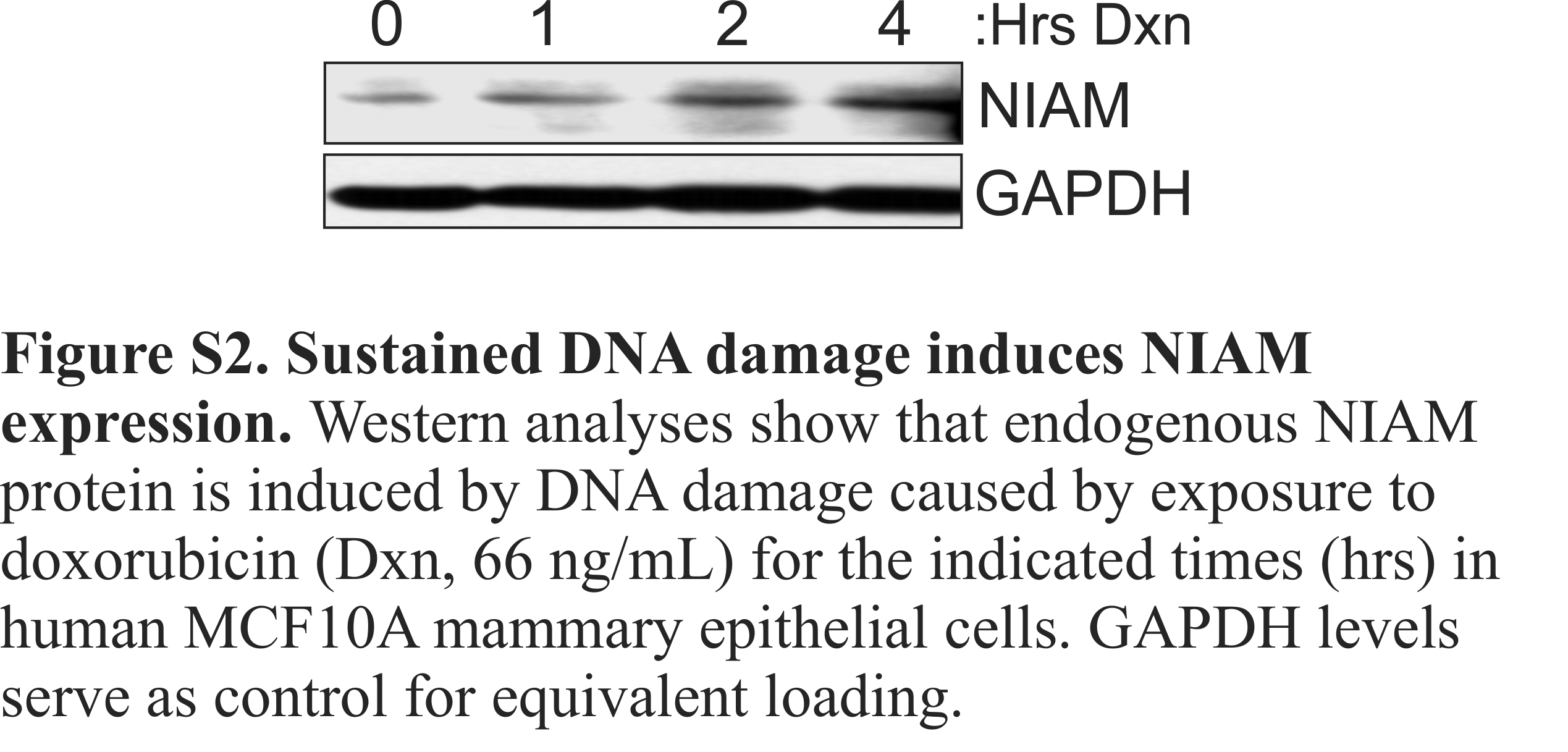

Supplement: Figure S2 — Sustained DNA damage induces NIAM expression. Western analyses show that endogenous NIAM protein is induced by DNA damage caused by exposure to doxorubicin (Dxn, 66 ng/mL) for the indicated times (hrs) in human MCF10A mammary epithelial cells. GAPDH levels serve as control for equivalent loading. (TIF) [file pone.0112126.s002.tif]
